# *ZmCom1* Is Required for Both Mitotic and Meiotic Recombination in Maize

**DOI:** 10.3389/fpls.2018.01005

**Published:** 2018-07-16

**Authors:** Yazhong Wang, Luguang Jiang, Ting Zhang, Juli Jing, Yan He

**Affiliations:** MOE Key Laboratory of Crop Heterosis and Utilization, National Maize Improvement Center of China, China Agricultural University, Beijing, China

**Keywords:** maize, meiosis, HR, DSB, COM1

## Abstract

CtIP/Ctp1/Sae2/Com1, a highly conserved protein from yeast to higher eukaryotes, is required for DNA double-strand break repair through homologous recombination (HR). In this study, we identified and characterized the COM1 homolog in maize. The *ZmCom1* gene is abundantly expressed in reproductive tissues at meiosis stages. In *ZmCom1*-deficient plants, meiotic chromosomes are constantly entangled as a formation of multivalents and accompanied with chromosome fragmentation at anaphase I. In addition, the formation of telomere bouquet, homologous pairing and synapsis were disturbed. The immunostaining assay showed that the localization of ASY1 and DSY2 was normal, while ZYP1 signals were severely disrupted in *Zmcom1* meiocytes, indicating that *ZmCom1* is critically required for the proper SC assembly. Moreover, RAD51 signals were almost completely absent in *Zmcom1* meiocytes, implying that COM1 is required for RAD51 loading. Surprisingly, in contrast to the *Atcom1* and *Oscom1* mutants, *Zmcom1* mutant plants exhibited a number of vegetative phenotypes under normal growth condition, which may be partly attributed to mitotic aberrations including chromosomal fragmentation and anaphase bridges. Taken together, our results suggest that although the roles of *COM1* in HR process seem to be primarily conserved, the *COM1* dysfunction can result in the marked dissimilarity in mitotic and meiotic outcomes in maize compared to Arabidopsis and rice. We suggest that this character may be related to the discrete genome context.

## Introduction

Meiosis is a highly conserved process producing haploid germ cells from diploid progenitors and is essential for all sexually reproductive organisms. It includes one round of DNA replication followed by two sequential rounds of cell division containing meiosis I and meiosis II (Zickler and Kleckner, 1999). During meiosis I, crossovers (COs) are formed to ensure the accurate segregation of homologous chromosomes (Mercier et al., 2015; Gray and Cohen, 2016). Homologous recombination (HR) is a prerequisite to the generation of COs. In plants, meiotic recombination is initiated by the programmed introduction of double-strand breaks (DSBs) mediated by SPO11, a conserved type II topoisomerase, and several accessory proteins (Keeney et al., 1997). The resulting DSB ends are resected by a protein complex, MRX/N (Mre11-Rad50-Xrs2/Nbs1) and Sae2/Com1/CtIP/Ctp1, to generate extended single-stranded DNA (ssDNA) overhangs, which are subsequently stabilized by replication protein A (RPA) (Borde, 2007). Next, RPA is displaced by RAD51 and DMC1 to form nucleoprotein filaments that can facilitate homologous pairing and single-end invasion of a non-sister chromatid resulting in the formation of joint molecule (JM) intermediates (Hunter and Kleckner, 2001). Ultimately, these events give rise to either COs or non-crossovers (NCOs) (Allers and Lichten, 2001).

The evolutionarily conserved MRX/N complex functions as one of the critical guardians of genome integrity in eukaryotes and is required for DNA damage repair, cell-cycle checkpoint and telomere maintenance during both mitosis and meiosis (Daoudal-Cotterell et al., 2002; Borde, 2007; Amiard et al., 2010). The three proteins (Mre11, Rad50, and Nbs1/Xrs2) in MRX/N complex play distinct roles. Mre11 specifies 3′ to 5′ exonuclease activity on the double-stranded DNA and endonuclease activity on the single-stranded DNA as well as limited helicase activity (Puizina et al., 2004; Altun, 2008). Rad50 has two long coiled-coil domains that interact with one another to form a head-to-tail dimer to enable the binding of Mre11 and DNA (Carney et al., 1998; Hopfner et al., 2002). NBS1 is phosphorylated by ATM to link the detection of DSBs to signaling events (Waterworth et al., 2007). Null mutations in genes encoding any component of MRX/N complex result in lethality in mammals (Paull and Gellert, 1998), whereas Arabidopsis *mre11* and *rad50* mutants are viable but fully sterile (Daoudal-Cotterell et al., 2002; Puizina et al., 2004; Samanic et al., 2013). In contrast, the loss-of-function of Arabidopsis *NBS1* displays normal growth under standard conditions and shows no defects in fertility (Waterworth et al., 2007). In addition, Arabidopsis mutants defective MRX/N complex in exhibit distinct hypersensitivity to various genotoxic stresses, reflecting both common and unique features of each component of MRX/N complex acting in the different spectrum of DNA lesions and mechanism of their repair (Vannier et al., 2006; Cassani et al., 2018).

As a cofactor for MRX/N, the mammalian CtIP and its fission yeast (Ctp1), budding yeast (Sae2), and plant (Com1) orthologs play the multifunctional roles in directing DSB repair pathway choice and modulate repair activities (McKee and Kleckner, 1997; Prinz et al., 1997; Baroni et al., 2004; Chen et al., 2005; Lengsfeld et al., 2007; Limbo et al., 2007; Penkner et al., 2007; Sartori et al., 2007; Uanschou et al., 2007; Lee-Theilen et al., 2010; Ji et al., 2012). The plant homolog of *CtIP/Ctp1/Sae2/Com1* was first identified in Arabidopsis (Uanschou et al., 2007) and later in rice (Ji et al., 2012). *Atcom1* and *Oscom1* mutant plants exhibit normal vegetative growth but complete male and female sterility (Uanschou et al., 2007; Ji et al., 2012). Cytological investigations revealed that meiosis is severely inhibited, due to the defective homologous pairing and massive chromosome fragmentation (Uanschou et al., 2007; Ji et al., 2012). These studies demonstrate that the function of *Com1* homolog in controlling DSB resection is conserved in plants as in other organisms.

In contrast to Arabidopsis and rice, maize has a large genome (ca. 2.3 Gb) and fairly complex genome organization. Here, we characterize the *Com1* in maize using a reverse genetic approach. Our results demonstrate that *ZmCom1* is essential for DSB repair and HR, establishing the telomere bouquet and SC assembly in maize meiosis. We also show that *ZmCom1* is required for mitosis to occur normally in vegetative cells. These results imply that although the roles of *Com1* in DSB repair seem to be fundamentally conserved in diverse plant species, the precise behavior of *Com1* may vary in the different plant organisms.

## Materials and Methods

### Plant Materials and Genotyping

Uniform*Mu* mutant lines, UF*Mu*-01240 (*Zmcom1-1*) and UF*Mu*-09026 (*Zmcom1-*2) induced by Robertsons *Mutator* transposons in the uniform W22 inbred line were obtained from Maize Stock Center and backcrossed with the W22 inbred line four times before the further analysis. All plants were grown in field or greenhouse in 2014–2017 under the normal growth condition. Genomic DNA extraction and genotyping were conducted as described previously (Li et al., 2013). To confirm a presence of the *Mutator* insertion, genomic DNA of both mutant lines was amplified with the primer pair of *Mu*TIR and COM1-L2 (**Supplementary Table S1**) and then PCR product was subject to Sanger sequencing.

### Observation of Pollen Viability

Pollen viability was assessed by Alexander staining using previously described methods (Alexander, 1969; Johnson-Brousseau and McCormick, 2004). Anthers were collected from the wild type and *Zmcom1* mutants during anthesis stage. Pollen grains were dissected out of anthers in Alexander solution and analyzed under Leica EZ4 HD. The pictures of strained pollen grains were taken using the microscope (Leica DM2000 LED).

### cDNA Cloning, RT-PCR and RT-qPCR Analysis

Total RNA was extracted from roots, stems, leaves, developing embryos (16 days after pollination), endosperm (16 days after pollination), meiotic ears as well as anthers of B73 plants and young ear of *Zmcom1* plants, and was then reverse-transcribed into cDNA by TaKaRa kits following manufacturer’s instructions. The full-length cDNA was generated using the TransStart FastPfu Fly DNA Polymerase kit (TransGen). PCR primers used for RT-PCR and RT-qPCR are listed in **Supplementary Table S1**. The maize *UBIQUITIN* gene was used as a control standard for all tissues. RT-qPCR analysis was performed using the 7500 Fast Real-Time PCR System (Applied Biosystems).

### Subcellular Localization

The coding sequence of *ZmCOM1* was amplified with the primer pair PCUN-COM1 (**Supplementary Table S1**) and sub-cloned into of the pCUN+GFP vector using the *Bam*HI and *Spe*I sites to create an ORF encoding an EGFP fusion protein driven by the 35S promoter. Mesophyll protoplasts were isolated from the second leaves of 2-week-old etiolated B73 seedlings according to the method described previously (Yoo et al., 2007) and transformed with the prepared plasmids using the polyethylene glycol (PEG) mediated transformation method as previously described (Yoo et al., 2007). The protoplasts were cultured at 25°C in the dark for 18 h and observed under a confocal laser scanning microscope (Leica sp5).

### Preparation of Mitotic Chromosome Spreads

Chromosome spreads were prepared as described previously (Kato et al., 2004). Kernels were soaked for a night in sterile water before germinating at 30°C for 2–3 days. Root tips of 1–2 cm length were dissected and fixed in a 3:1 mixture of 95% ethanol: glacial acetic acid for 30 min in a vacuum environment and finally stored in 70% ethanol at -20°C until use. After washing in water on ice, the root apical meristem containing dividing cells was dissected and digested in 50 μl enzyme mix containing 1% pectolyase Y23 (ICN) and 2% cellulase Onozuka R-10 (Yakult Pharmaceutical, Tokyo) for 65 min at 37°C. After digestion, the root sections were washed in ice-cold distilled water and then briefly washed in 70% ethanol for three times. The root sections were carefully broken using a needle and vortexed at maximum speed in 75% ethanol for 30 s at room temperature to separate cells from each other. Cells were collected at the bottom of the tube by centrifugation and resuspended in 100% glacial acetic acid solution. Ten microliter of the cell suspension was dropped onto glass slides in a box lined with wet paper towels and dried slowly.

### Preparation of Meiotic Chromosome Spreads

Chromosome spreads were prepared from tassels fixed in Carnoy’s solution (3:1 ethanol: acetic acid, v/v). After infiltration for 30 min at room temperature, the tassels were stored in 75% ethanol at 4°C until observation. Squashes were made in a drop of 45% acetic acid. The microscope slides were frozen in liquid nitrogen and the coverslips were removed immediately. The slides were then dehydrated through an ethanol series (70% to 90% to 100%) for 5 min each and air dried. The chromosomes were stained with 4′, 6-diamidino-2-phenylindole (DAPI) in an antifade solution (Vector, H-1200, CA, United States). Images were captured using a Ci-S-FL microscope (Nikon, Tokyo) equipped with a DS-Qi2 Microscope Camera system.

### FISH Analysis

The FISH procedure was performed as described previously (Li and Arumuganathan, 2001; Cheng, 2013). Plasmids carrying 5S rDNA repeats (pTa794) or the telomere-specific repeats (pAtT4) were used as FISH probes (Richards and Ausubel, 1988; Ji et al., 2012). The 5S rDNA-specific and telomere-specific probes were individually labeled with digoxigenin by nick translation (Roche, Cat.No.11745808910) and detected with a fluorescein isothiocyanate (FITC) conjugated anti-digoxigenin antibody (Vector Laboratories). The chromosomes were counterstained with DAPI in Vectashield antifade solution (Vector laboratories). Chromosome spreads were observed under a Ci-S-FL fluorescence microscope (Nikon) and captured with a DS-Qi2 Microscope Camera.

### Fluorescence Immunolocalization

Young anthers at the meiotic prophase (∼1.5–2.5 mm, Zhang et al., 2014) were fixed with 4% (w/v) paraformaldehyde in 1 × Buffer A for 30 min at room temperature (25°C), washed in 1 × Buffer A at room temperature and stored in 1 × Buffer A at 4°C for several months. The procedure of immunolocalization was performed as described previously (Pawlowski et al., 2003; Cheng, 2013). All primary (ASY1, DSY2, ZYP1, and RAD51) and secondary antibodies were used at a dilution of 1:100. The images were viewed with software NIS-Elements to generate 2D projected images. Surface rendered images were colored by the ImageJ software through the Merge Channels.

## Results

### Identification of *ZmCom1* and Isolation of Its Mutants

**FIGURE 1:**
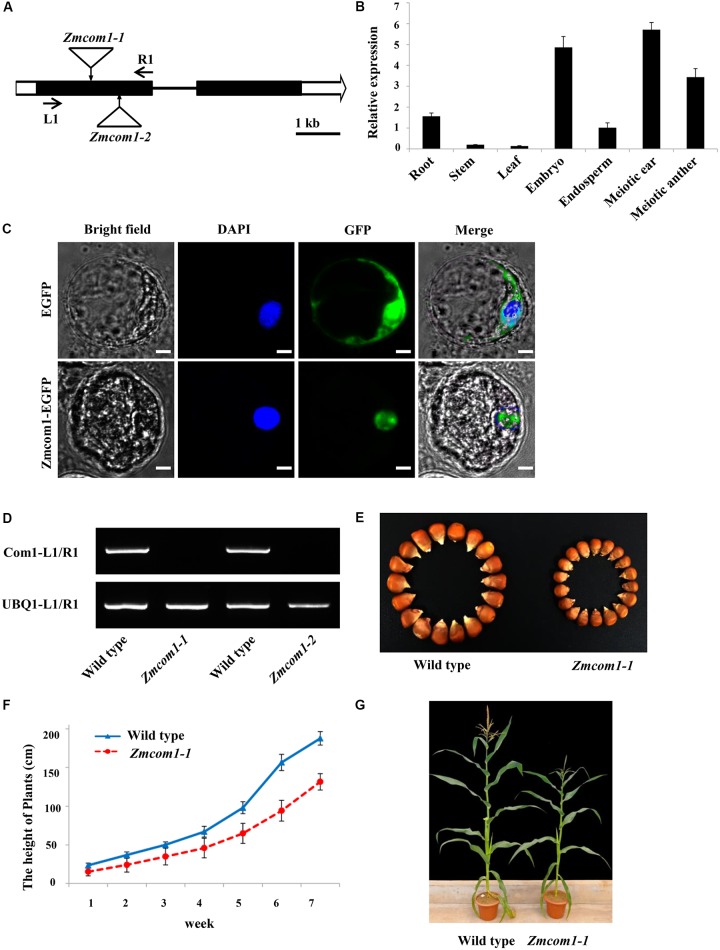
Characterization of the *ZmCom1* gene. **(A)** Diagram of genomic structures of the *ZmCom1* genes with Mutator transposon insertion sites marked with triangles. Bars indicate exons and lines represent introns. Scale bar = 1 kb. **(B)** RT-qPCR analysis of *ZmCom1* transcription in different tissues. Expression levels relative to the expression level of the UBQ1 gene are presented with standard errors. Values are means of three independent experiments. **(C)** ZmCom1 protein is localized to nucleus in maize protoplasts. Scale bar = 5 μm. **(D)** RT-PCR analysis of *ZmCom1* transcript in different genotypes. The maize *UBQ1* gene was used as an internal control. **(E)** Morphological comparison of mature seeds between wild type and *Zmcom1-1* mutant. **(F)** Growth-curve of plant height in wild type and *zmcom1-1* mutant plants. Values are means of 10 individual plants. **(G)** Morphological comparison of mature plants between wild type and *Zmcom1-1* mutant.

A BLASTP search using the rice Com1 amino acid sequence was conducted in the maize genome database (MaizeGDB) and only one candidate gene model (GRMZM2G076617) with significant similarity was identified. The cDNA sequence, which was redefined by rapid amplification of cDNA ends (RACE) PCR, contains 2,134 bp with an open reading frame of 1,668 bp. The gene has two exons and one intron (**Figure 1A**). The protein sequence consists of 555 amino acids and shows 62% of identity and 72% of similarity to OsCom1 (363/583 residues identical and 421/583 residues positive, **Supplementary Figure S1**). ZmCom1 protein harbors an N-terminal SMC-N domain and a C-terminal SAE2 superfamily domain, both of which conventionally exist in Com1 homologs of other organisms (**Supplementary Figure S1**). Phylogeny analyses revealed that Com1 homologs form two distinct clades reflecting the divergence between monocots and dicots (**Supplementary Figure S2**).

By means of quantitative RT-PCR, we examined the tissue-specific expression pattern of *ZmCom1*. We found that *ZmCom1* is expressed most highly in meiotic ears and anthers, as well as in developing embryo, less in root and endosperm, and extremely low in leaf and stem (**Figure 1B**). These results support the function of *ZmCom1* in meiosis and mitosis. To elucidate the cellular localization of *ZmCom1*, we induced expression of the ZmCom1 fused to the EGFP under the control of the CaMV35S promoter in maize protoplasts. The GFP signal was revealed in nuclei (**Figure 1C**).

Two independent *Mutator* transposon insertion lines, UF*Mu*-01240 (*Zmcom1-1*) and UF*Mu*-09026 (*Zmcom1-2*), were identified in the public maize *Mutator* line database (Harper et al., 2016). By conducting locus-specific PCR amplification followed by Sanger sequencing, we confirmed that both insertion sites are within the first exon of *ZmCom1* (**Figure 1A**). RT-PCR with primers flanking the *Mutator* insertion sites failed to detect the *ZmCom1* transcripts (**Figure 1D**), indicating that both mutants are null. The heterozygous alleles of both mutants did not exhibit any obvious defects during either the vegetative or reproductive stages in comparison to the wild type. However, we constantly observed a proportion of small kernels in the offspring of self-pollinated heterozygous plants for both mutations (**Figure 1E** and **Supplementary Figure S3A**), and the ratio of small to normal seeds was not significantly different from the expected 1:3 ratio (Chi-square test, *P* > 0.05; **Supplementary Table S2**). More importantly, PCR analyses confirmed that these small kernels co-segregated with homozygous *Zmcom1* genotype (**Supplementary Figure S4**), indicating that the loss-of-function of *ZmCom1* has an effect on maize seed development. The overall statue of *Zmcom1* plants seemed to be comparable to wild type, but obvious dwarf phenotype started appearing from first weeks until the maturity (**Figures 1F,G** and **Supplementary Figures S3B,C**).

**FIGURE 2:**
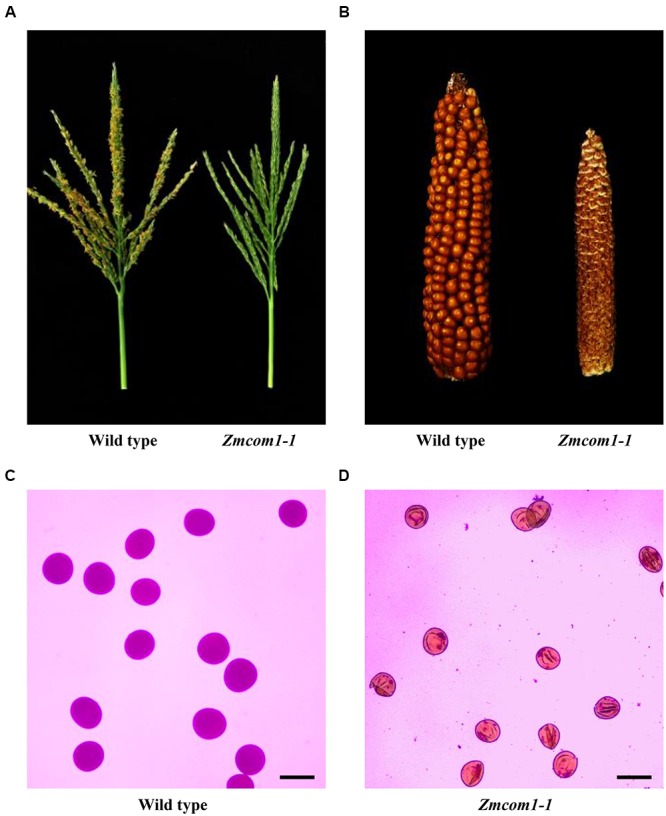
Male and female sterility of *Zmcom-1* mutant plants. **(A)** Comparison of a wild type tassel and a *Zmcom1-1* tassel at the flowering stage. **(B)** Comparison of a wild type ear and a *Zmcom1-1* ear. **(C)** Normal pollen grains of the wild type. Scale bar = 100 μm. **(D)** Complete sterile pollen grains of the *Zmcom1-1* plant. Scale bar = 100 μm.

The *Zmcom1* plants reached the anthesis stage at the same time as wild type but were completely male sterile (**Figure 2A** and **Supplementary Figure S3D**). When pollinated with normal pollen grains from the wild type, the *Zmcom1* plants could not set any seeds (**Figure 2B** and **Supplementary Figure S3E**), suggesting that megagametogenesis was aborted. To investigate the male sterility, *Zmcom1* and wild type pollen grains were stained with the Alexander solution (**Figures 2C,D** and **Supplementary Figure S3F,G**). Pollen grains from the wild type were round (**Figure 2C** and **Supplementary Figure S3F**), while those from *Zmcom1* plants were empty and shrunken (**Figure 2D** and **Supplementary Figure S3G**), indicating that microspore development was also aborted. Taken together, these results indicate that the disruption of *ZmCom1* gene leads to defects in both vegetative and reproductive development.

### Abnormal Chromosome Behavior in *Zmcom1*

**FIGURE 3:**
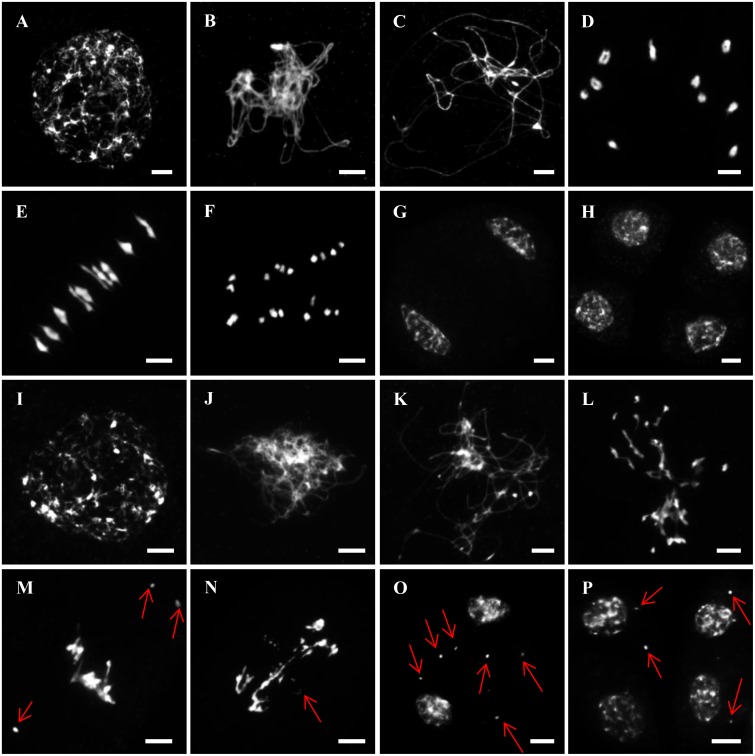
Male meiosis in wild type and *Zmcom1-1*. **(A–H)** Meiosis in wild type. **(A)** Leptotene; **(B)** Zygotene; **(C)** Pachytene; **(D)** Diakinesis; **(E)** Metaphase I; **(F)** Anaphase I; **(G)** Dyad; **(H)** Tetrads. Scale bars = 10 μm. **(I–P)** Meiosis in *Zmcom1-1*. **(I)** Leptotene; **(J)** Zygotene; **(K)** Pachytene; **(L)** Diakinesis; **(M)** Metaphase I; **(N)** Anaphase I; **(O)** Dyad; **(P)** Tetrads. The red arrows pointed out the chromosomal fragments and abnormal bridges. Scale bars = 10 μm.

To establish the cause of sterility in the *Zmcom1* mutant, we examined the meiotic chromosome behavior in pollen mother cells (PMCs) of both wild type and *Zmcom1-1* plants using 4′,6-diamidino-2-phenylindole (DAPI) staining (**Figure 3**). In the wild type, the chromosomes appeared as thin threads at leptotene (**Figure 3A**), and homologous chromosomes underwent pairing and synapsis during zygotene (**Figure 3B**). At pachytene, homologous chromosomes completed synapsis and chromosomes appeared as thick threads (**Figure 3C**). During diakinesis, chromosomes became condensed, and 10 short bivalents connected by chiasmata were observed in nuclei (**Figure 3D**). At metaphase I, all 10 bivalents aligned in an orderly manner on the equatorial plate (**Figure 3E**), and homologous chromosomes separated and migrated to opposite poles at anaphase I (**Figure 3F**). Finally, all chromosomes reached the two poles at telophase I to form regular dyads (**Figure 3G**). During meiosis II, sister chromatids separated from each other, ultimately giving rise to tetrad (**Figure 3H**).

In *Zmcom1* mutant, meiotic chromosomes behaved normally at leptotene (**Figure 3I** and **Supplementary Figure S5A**). However, the chromosomes remained as single threads and did not pair with their homologs during zygotene and pachytene (**Figures 3J,K** and **Supplementary Figures S5B,C**). Irregularly shaped univalents were scattered throughout the entire nucleus during diakinesis (**Figure 3L** and **Supplementary Figure S5D**). At metaphase I, chromosomes intertwined into a block, and chromosome fragments appeared as small dots (**Figure 3M** and **Supplementary Figure S5E**). During anaphase I, these entangled chromosomes separated, resulting in unequal segregation of chromosomes to the opposite poles. Chromosome bridges were constantly observed and chromosome fragments remained at the equatorial plate (**Figure 3N** and **Supplementary Figure S5F**). Although most chromosomes had arrived in the two poles at telophase I, many lagging fragments were still randomly scatted within the nucleus (**Figure 3O** and **Supplementary Figure S5G**). After the second division, abnormal tetrads with several micronuclei were eventually generated (**Figure 3P** and **Supplementary Figure S5H**). Therefore, we concluded that the sterility of the *Zmcom1* mutant may be caused by deficiency in homologous chromosome pairing, synapsis, and profound chromosomal fragmentation.

### Defective Telomere Bouquet Formation and Homologous Pairing in *Zmcom1*

**FIGURE 4:**
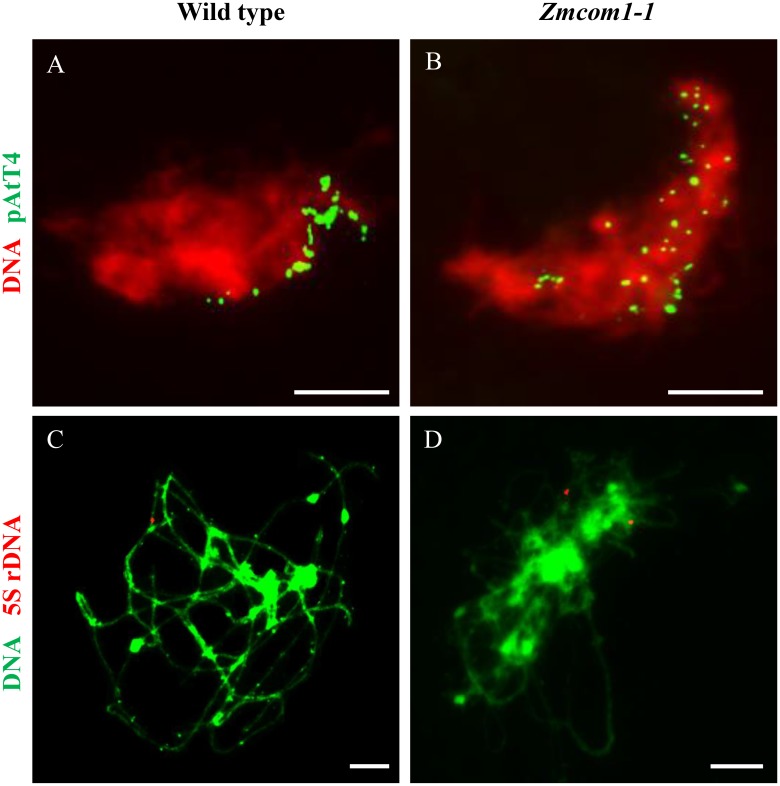
*ZmCom1* is essential for bouquet formation and homologous pairing. **(A,B)** Bouquet formation analysis using FISH with the telomere-specific pAtT4 probe in in the wild type **(A)** and *Zmcom1-1* **(B)**. Scale bars = 10 μm. **(C,D)** Homologous pairing analysis using FISH with 5S rDNA probe in in the wild type **(C)** and *Zmcom1-1* **(D)**. Scale bars = 10 μm.

Telomere bouquet clustering, a particular event in early prophase I, may facilitate the initiation of homologous pairing (Golubovskaya et al., 2002; Harper et al., 2004; Klutstein et al., 2015). To explore the pairing defects in *Zmcom1*, we conducted fluorescent *in situ* hybridization (FISH) analysis using the telomere-specific probe in wild type and *Zmcom1* meiocytes (**Figures 4A,B** and **Supplementary Figure S6A**). In wild type meiocytes (*n* = 46), 97.8% of the telomere signals attached to the nuclear envelop and were clustered at early zygotene stage, displaying a typical telomere bouquet formation (**Figure 4A**). However, in *Zmcom1* meiocytes (*n* = 55 and 41 for *Zmcom1-1* and *Zmcom1-2*, respectively), telomeres did not cluster within a certain region but scattered throughout the nucleus (**Figure 4B** and **Supplementary Figure S6A**), indicating that the telomere bouquet formation was defective in the *Zmcom1* mutants.

To further determine chromosome pairing behavior in *Zmcom1* mutant, we performed FISH experiments using 5S rDNA as probes in wild type and *Zmcom1* meiocytes (**Figures 4C,D** and **Supplementary Figure S6B**). 5S rDNA is a tandemly repetitive sequence that only locates on the distal regions of the long arm of chromosome 2 (Li and Arumuganathan, 2001). In wild type meiocytes (*n* = 32), only one 5S rDNA signal was detected at pachytene stage (**Figure 4C**), indicating that two homologous chromosomes had been well paired. In contrast, two separate 5S rDNA signals were detected in *Zmcom1* meiocytes (*n* = 38 and 27 for *Zmcom1-1* and *Zmcom1-2*, respectively) (**Figure 4D** and **Supplementary Figure S6B**). These results further confirmed that homologous chromosome paring was deficient in *Zmcom1*.

### Normal Axial Element Installation but Deficient Central Element Installation in *Zmcom1*

**FIGURE 5:**
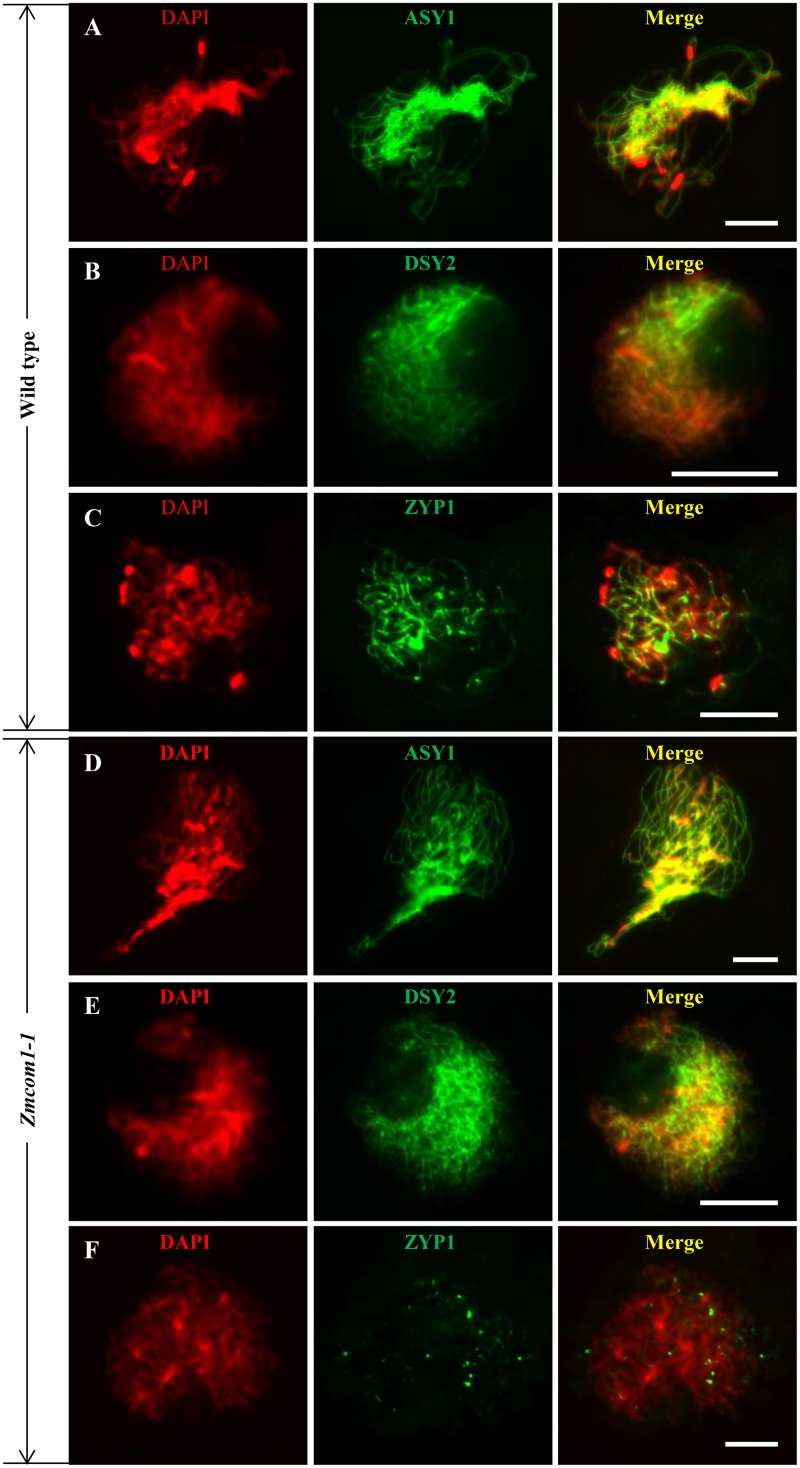
Immunolocalization of ASY1, DSY2, and ZYP1 antibodies in the wild type and *Zmcom1-1*. **(A–C)** ASY1 **(A)**, DSY2 **(B)**, and ZYP1 **(C)** on prophase I chromosomes in the wild type. DAPI staining is used to indicate the chromosomes. Scale bars = 10 μm. **(D–F)** ASY1 **(D)**, DSY2 **(E)**, and ZYP1 **(F)** on prophase I chromosomes in *Zmcom1-1*. Scale bars = 10 μm.

The SC consists of two parallel lateral elements (LEs – former axial elements – AEs) and one central element (CE). To investigate whether the SC was properly assembled in *Zmcom1-1*, we conducted immunostaining analysis using antibodies against the maize SC components ASY1, DSY2, and ZYP1. ASY1, a homolog of rice PAIR2, is the AE protein which plays pivotal roles in bouquet formation, homologous pairing and the SC assembly (Sanchez-Moran et al., 2008; Wang et al., 2011). We found that ASY1 distribution on chromosomes in *Zmcom1-1* meiocytes (*n* = 42) was similar to that in wild type meiocytes (*n* = 53) (**Figures 5A,D**). DSY2, a homolog of rice PAIR3 and Arabidopsis ASY3, acts as a structural protein to connect the AE/LEs to the CE for the SC assembly (Wang et al., 2011; Ferdous et al., 2012; Lee et al., 2015). In *Zmcom1-1* meiocytes (*n* = 56), DSY2 also loaded regularly onto chromosomes during zygotene, and did not show any difference from the wild type (*n* = 47) (**Figures 5B,E**). We also investigated the installation of the AE components in *Zmcom1-2*, which was similar to those of *Zmcom1-1* (**Supplementary Figures S7A,B**). Therefore, we conclude that the loss-of-function of *ZmCom1* has no significant effect on the installation of the AEs.

ZYP1, a transverse filament protein, constitutes the CE of the SC in maize (Higgins et al., 2005; Wang et al., 2010; Barakate et al., 2014). In the wild type, ZYP1 was first detected as discontinuous foci at the leptotene stage during zygotene, and it gradually formed discontinuous linear signals. At pachytene, ZYP1 signals were aligned perfectly along the entire chromosome length (*n* = 35; **Figure 5C**). In the *Zmcom1-1* meiocytes, ZYP1 signals could not elongate to form linear signals and only present as punctate signals (*n* = 63; **Figure 5F**). We also investigated the installation of the CE component in *Zmcom1-2*, which was similar to those of *Zmcom1-1* (**Supplementary Figure S7C**). Taken together, we conclude that the SC assembly is deficient in *Zmcom1*.

### *ZmCom1* Is Critical for DSB Repair

**FIGURE 6:**
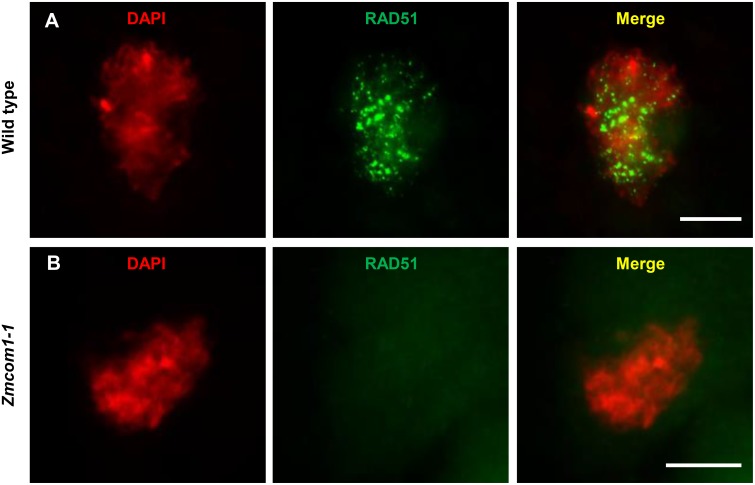
Immunolocalization of RAD51 antibodies in the wild type and *Zmcom1-1* meioctyes at zygotene. **(A)** Wild type, **(B)** *Zmcom1-1* mutant. DAPI staining is used to indicate the chromosomes. Scale bars = 10 μm.

The chromosome fragmentation observed in *Zmcom1-1* meiocytes suggests that DSBs could still maintain due to loss-of-function of *ZmCom1*, as it was observed in *Oscom1* (Ji et al., 2012) and *Atcom1* (Uanschou et al., 2007). To ascertain whether defective homologous pairing and synapsis in *Zmcom1* were correlated with the improper DSB repair, the RAD51 immunostaining experiment was performed in wild type and *Zmcom1-1* meiocytes. Loading of RAD51 onto the ssDNA serves as a cytological marker for DSB repair via HR in different organisms (Pawlowski et al., 2003). In the wild type zygotene meiocytes, a substantial number of RAD51 foci was observed (*n* = 33, **Figure 6A**). In contrast, a parallel experiment did not detect any RAD51 foci in *Zmcom1-1* (*n* = 49, **Figure 6B**) or *Zmcom1-2* meiocytes (*n* = 36, **Supplementary Figure S8**). These results indicate that *ZmCom1* is required for the proper recruitment/loading of RAD51 onto the chromosomes and further demonstrate a serious defect in DSB repair in *Zmcom1*.

### Somatic Aberrations in *Zmcom1*

**FIGURE 7:**
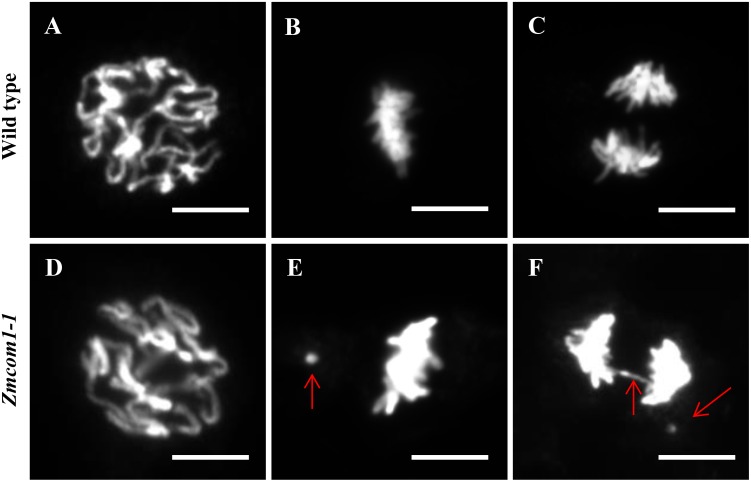
Genome instability in mitotic cells from *Zmcom1-1* plants. **(A–C)** Wild type. **(A)** Prophase; **(B)** Metaphase; **(C)** Anaphase. Scale bars = 10 μm. **(D–F)** *Zmcom1-1*. **(D)** Prophase; **(E)** Metaphase; **(F)** Anaphase. Lagging.

Unlike *Atcom1* and *Oscom1*, *Zmcom1* exhibited vegetative aberrations under standard growth conditions. To explore whether and how the loss-of-function of *ZmCom1* influences the mitotic process, we assessed the frequency of chromosomal instability in root apical meristem for wild type and *Zmcom1* plants. At prophase, there was no obvious deviation between the wild type and *Zmcom1-1* (**Figures 7A,D**). However, we consistently observed an increased occurrence of acentric fragments at mitotic metaphase in *Zmcom1-1* (10.5%, *n* = 238; **Figure 7E** and **Table 1**) compared to that in the wild type (0.3%, *n* = 323; **Figure 7B** and **Table 1**). Later, ∼12.8% of mitotic cells had bridges or chromosome fragments in *Zmcom1-1* anaphase (*n* = 258; **Figure 7F** and **Table 1**), significantly higher than ∼0.3% of that in the wild type (*n* = 351; **Figure 7C** and **Table 1**). We also investigated the mitotic process in *Zmcom1-2*, which was similar to that of *Zmcom1-1* (**Supplementary Figure S9**). These results suggest that *Zmcom1* mutant suffers somatic chromosomal destabilization even under the normal growth condition.

**Table 1:** Genome Instabilities in wild type and *Zmcom1* mitotic cells.

	Metaphase		Anaphase		
	No. of cell scored	No. (percentage) of cell appearing fragment	No. of cell scored	No. (percentage) of cell appearing bridges	No. (percentage) of cell appearing fragments
Wild type	323	1 (0.3%)	351	1 (0.3%)	0
*Zmcom1-1*	238	25 (10.5%)	258	18 (7.0%)	15 (5.8%)
*Zmcom1-2*	276	28 (10.1%)	231	15 (6.5%)	13 (5.6%)

## Discussion

### Role of *ZmCom1* in Maize Meiosis

The conserved roles of CtIP/Ctp1/Sae2/Com1 in meiosis have been identified in several organisms. Consistent with this, in the present study we show that the loss-of-function of the *ZmCom1* gene leads to chromosome fragmentation and defects in homologous pairing and synapsis during meiosis, indicating that also in maize, *Com1* is an essential element in DSB repair. However, the precise effect of Com1 homolog on chromosome behavior differs from other plant organisms.

Telomere bouquet formation, a specialized arrangement of chromosomes during early prophase of meiosis in which telomeres are clustered on the nuclear envelope, has been observed in some plant species, animals and fungi (Niwa et al., 2000; Harper et al., 2004; Ding et al., 2007). Numerous maize mutants exhibit the defective bouquet including *pam1* (Golubovskaya et al., 2002), *dy* (Murphy and Bass, 2012), *dsy1* (Bass et al., 2003), *afd1* (Golubovskaya et al., 2006), and *phs1* (Pawlowski et al., 2004), as well as the rice *pair3* (Wang et al., 2011) and *zygo1* (Zhang et al., 2017). Meanwhile, all these mutants also show the concurrent abnormality in homologous pairing, suggesting that the proper bouquet formation is a key event to facilitate homologous chromosome pairing (Zhang et al., 2017).

It is interesting that, the telomere bouquet formation is unaffected in the rice *com1* mutant and the telomere clustering is indistinguishable from that in the wild type (Ji et al., 2012), whereas in the maize *com1* mutants, a typical telomere clustering was never observed indicating that the Zm*Com1* gene is critically required for bouquet formation. Therefore, the remarkable difference between *ZmCom1* and *OsCom1* in mediating bouquet formation highlights the questions of why and how such character is conferred in different plant species. Also, the other intriguing question raised is whether the participation of *ZmCom1* in bouquet formation is restricted to its own character, or other members of MRN complex are also involved. Those questions would be of great interest in future studies.

Chromosome fragmentation and entanglements is a typical phenomenon observed in mutants deficient in DSB repair machinery. Our data showed that the *Zmcom1* mutant phenotype is similar to that of the *Oscom1* and *Atcom1* mutants, as well as other related mutants such as *Atmre11* (Samanic et al., 2013), *Atrad50* (Vannier et al., 2006), *Osxrcc3* (Zhang et al., 2015), and *Osrad51c* (Tang et al., 2014). However, chromosome segregation and the integrity of the tetrads seems to be less severe in *Zmcom1* compared to the *Oscom1* or *Atcom1* mutants. A simple explanation for this dissimilarity could be that the alternative DSB repair pathway, such as non-homologous end-joining (NHEJ) or microhomology-mediated end-joining (MMEJ) (McVey and Lee, 2008; Shrivastav et al., 2008), may be more actively stimulated in the absence of HR pathway in maize. In this scenario, *ZmCom1* act as a regulator to balance the different DSB repair pathways, a mechanism suggested in the previous studies (Ji et al., 2012). Therefore, it would be worth to investigate the meiotic consequences after combining mutation in *ZmCom11* with mutations in the genes involved in NHEJ and MMEJ pathway, which are largely unexplored yet in maize.

### Role of *ZmCom1* in Maize Mitosis

Unrepaired DSBs are one of the most lethal types of DNA damage and highly threaten on chromosome stability and cell survival (Edlinger and Schlögelhofer, 2011). Beside the programmed induction during meiosis, DSBs can be triggered by both endogenous (e.g., transposition events of transposable elements (TEs), errors of oxidative metabolism, stalled, or collapsed replication forks) and exogenous sources (e.g., ionizing radiation or genotoxic stresses) in the vegetative growth period. Organisms have evolved two major pathways, HR and NHEJ, for repairing DSBs and maintaining genome integrity. Coordinated with MRX/N complex, CtIP/Ctp1/Sae2/Com1 plays a critical role in HR. Therefore it is not surprised to find that the mutation in those genes will result in the increased sensitivity toward various genotoxic stresses. Indeed, *Atcom1* mutants showed the retarded development of true leaves after treatment with mitomycin C (Uanschou et al., 2007). Meanwhile, without the special treatment, *Atcom1* mutant plants grew well, and did not show any vegetative phenotypes compared to the wild type. This is also the case for *Oscom1* mutant plants. Those results suggest that under the normal condition, the endogenous DSBs can be efficiently repaired in spite of lack of intact *Com1*–dependent HR in both Arabidopsis and rice. However, we observed some mitotic and vegetative abnormalities in *Zmcom1* mutants when plants were grown under standard environmental conditions. As the appearance of those vegetative phenotypes seems to be unique for *Zmcom1* mutants, we speculate that it can be attributed to the special feature of maize chromosomes. In contrast to Arabidopsis and rice, maize has a large genome with over 85% of TEs (Schnable et al., 2009; Andres and Williams, 2017). Although the mobility for the majority of TEs would be principally silenced by DNA methylation, a fraction of TEs still remains the activity for jumping around genome and driving genetic evolution (Mirouze and Vitte, 2014). In this context, maize genome may suffer from a greater frequency of transposition-derived DSBs compared to rice and Arabidopsis. Alternatively, alike mammalian cells(Feng et al., 2016), the pervasive distribution of repetitive element on maize chromosomes may have a tendency to cause replication fork stalling and subsequent collapse of stalk fork, and can induce replication-associated DSBs (Nikolov and Taddei, 2016). In both scenarios, the Com1 activity is hypothetically required to maintain the genome integrity. Meanwhile, as both TE-transposition and the collapse of stalk fork frequently occur during the S-phase of the cell cycle, it would be also conceivable to explain how the disruption of *ZmCom*1 led to the abnormality in the seed development, a period when cells fast divide and proliferate.

## Author Contributions

YH conceived and supervised the project. YW, LJ, TZ, and JJ conducted the experiments. YW and YH prepared the manuscript. All authors read and approved the final manuscript.

## Conflict of Interest Statement

The authors declare that the research was conducted in the absence of any commercial or financial relationships that could be construed as a potential conflict of interest.

## Supplementary Materials

FIGURE S1 Protein sequence alignment of ZmCom1 and OsCom1. The proteins were aligned with CLUSTALW and image was made by MultAlign (http://multalin.toulouse.inra.fr/multalin/). Conserved (>90% conservation) amino acid residues are red, variable (<50% conservation) are blue. Species abbreviation: Zm, *Zea mays*; Os, *Oryza sativa*. The red and green underlines indicate the conserved SMC-N and SAE2 domain, respectively.

FIGURE S2 Neighbor-joining phylogeny reconstruction of Com1 homologs from different plant species. Numbers next to branches indicate posterior probability values. The scale indicates number of substitutions per site. Protein sequences were aligned using ClustalX (Jeanmougin et al., 1998) and phylogeny reconstruction was conducted using the online software (http://www.phylogeny.fr/, Dereeper et al., 2010). Species abbreviation: Zm, *Zea mays*; Os, *Oryza sativa*; Sb, *Sorghum bi*color; At, *Arabidopsis thaliana*; Bn, *Brassica napa*; Bd, *Brachypodium distachyon*; Hv, *Hordeum vulgare*; Ta, *Triticum aestivum*; Cs, *Camelina sativa*; Rs, *Raphanus sativus*; Es, *Eutrema salsugineum*.

FIGURE S3 Morphological comparison between wild type and *Zmcom1-2* mutant. **(A)** Morphological comparison of mature seeds between wild type and *Zmcom1-2* mutant. **(B)** Growth-curve of plant height in wild type and *Zmcom1-2* mutant plants. Values are means of 10 individual plants. **(C)** Comparison of Morphological comparison of mature plants between wild type and *Zmcom1-2* mutant. **(D)** Comparison of a wild type tassel and a *Zmcom1-2* tassel at the flowering stage. **(E)** Comparison of a wild type ear and a *Zmcom1-2* ear. **(F)** Normal pollen grains of the wild type. Scale bar = 100 μm. **(G)** Complete sterile pollen grains of the *Zmcom1-2* plant. Scale bar = 100μm.

FIGURE S4 PCR-based genotyping of seeds from self-propagated heterozygous *Zmcom1* plants. **(A)** F2 progeny of *Zmcom1-1*. 1–6: Seeds with normal size; 7–24: Seeds with small size. **(B)** F2 progeny of *Zmcom1-2*. 25–30: Seeds with normal size; 31–48: Seeds with small size.

FIGURE S5 Male meiosis in *Zmcom1-2*. **(A)** Leptotene; **(B)** Zygotene; **(C)** Pachytene; **(D)** Diakinesis; **(E)** Metaphase I; **(G)** Anaphase I; **(F)** Dyad; **(H)** Tetrads. The red arrows pointed out the chromosomal fragments and abnormal bridges. Scale bars = 10 μm.

FIGURE S6 The defective bouquet formation and homologous pairing in *Zmcom1-2.* **(A)** Bouquet formation analysis using FISH with the telomere-specific pAtT4 probe in *Zmcom1-2* (*n* = 41). Scale bars = 10 μm. **(B)** Homologous pairing analysis using FISH with 5S rDNA probe in in *Zmcom1-2* (*n* = 27). Scale bars = 10 μm.

FIGURE S7 Immunolocalization of ASY1, DSY2, and ZYP1 antibodies in *Zmcom1-2*. ASY1 (**A**, *n* = 23), DSY2 (**B**, *n* = 33), and ZYP1 (**C**, *n* = 37) on prophase I chromosomes in *Zmcom1-2*. Scale bars = 10 μm.

FIGURE S8 Immunolocalization of RAD51 antibodies in *Zmcom1-2* meioctyes (*n* = 36) at zygotene. DAPI staining is used to indicate the chromosomes. Scale bars = 10 μm.

FIGURE S9 Genome instability in mitotic cells from *Zmcom1-2* plants. **(A)** Prophase; **(B)** Metaphase; **(C)** Anaphase. Lagging chromosome fragments and anaphase bridges were highlighted by red arrows. Scale bars = 10 μm.

TABLE S1 Primers used in this study.

TABLE S2 Segregation ratio of small seeds versus normal seeds.
