# Supplementary figures and images for: ZmCom1 Is Required for Both Mitotic and Meiotic Recombination in Maize
Source: Front Plant Sci. 2018 Jul 16;9:1005. doi: 10.3389/fpls.2018.01005 (PMC6055016; doi:10.3389/fpls.2018.01005)

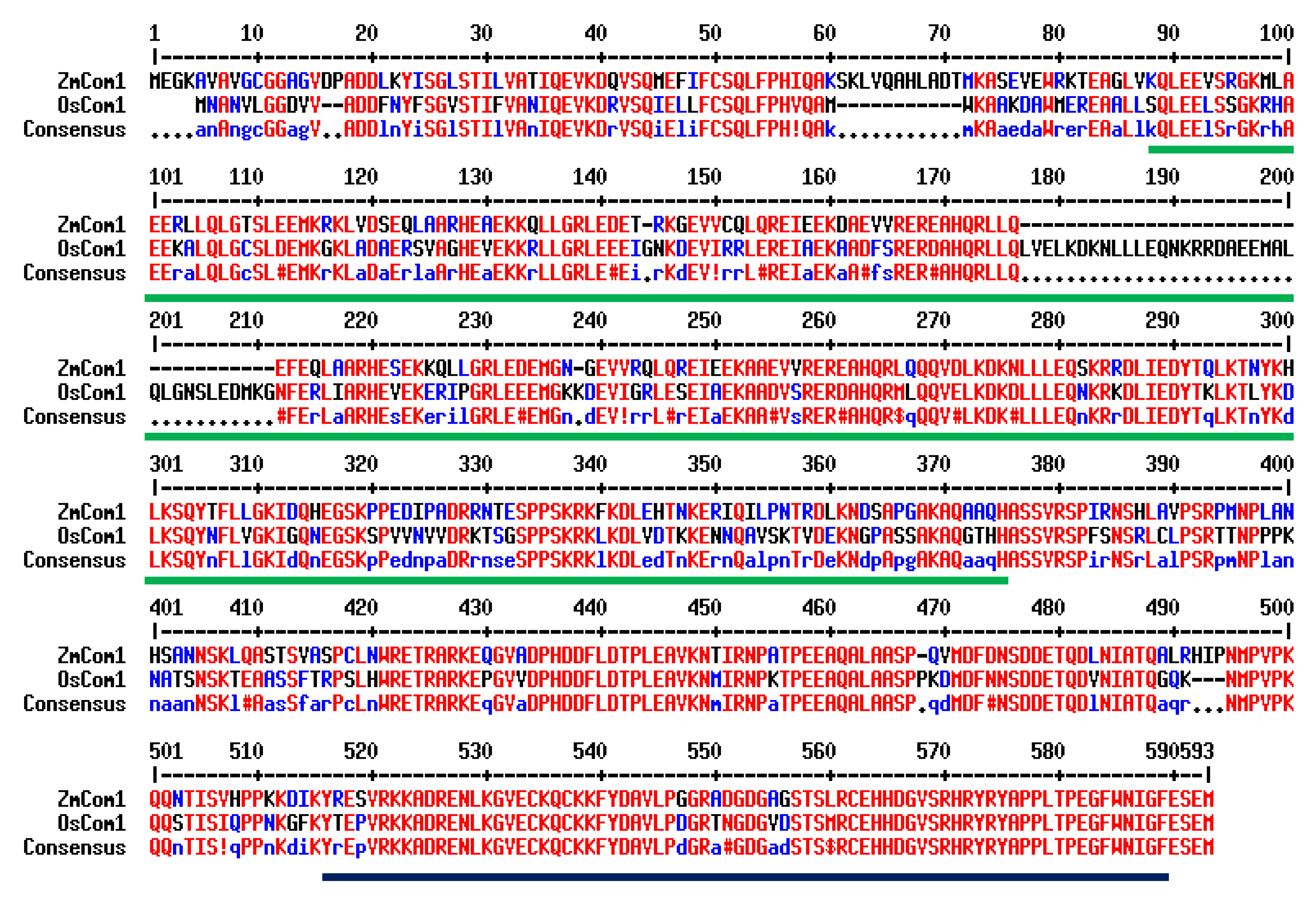

Supplement: FIGURE S1 — Protein sequence alignment of ZmCom1 and OsCom1. The proteins were aligned with CLUSTALW and image was made by MultAlign (http://multalin.toulouse.inra.fr/multalin/). Conserved (>90% conservation) amino acid residues are red, variable (<50% conservation) are blue. Species abbreviation: Zm, Zea mays; Os, Oryza sativa. The red and green underlines indicate the conserved SMC-N and SAE2 domain, respectively. [file Image_1.TIF]

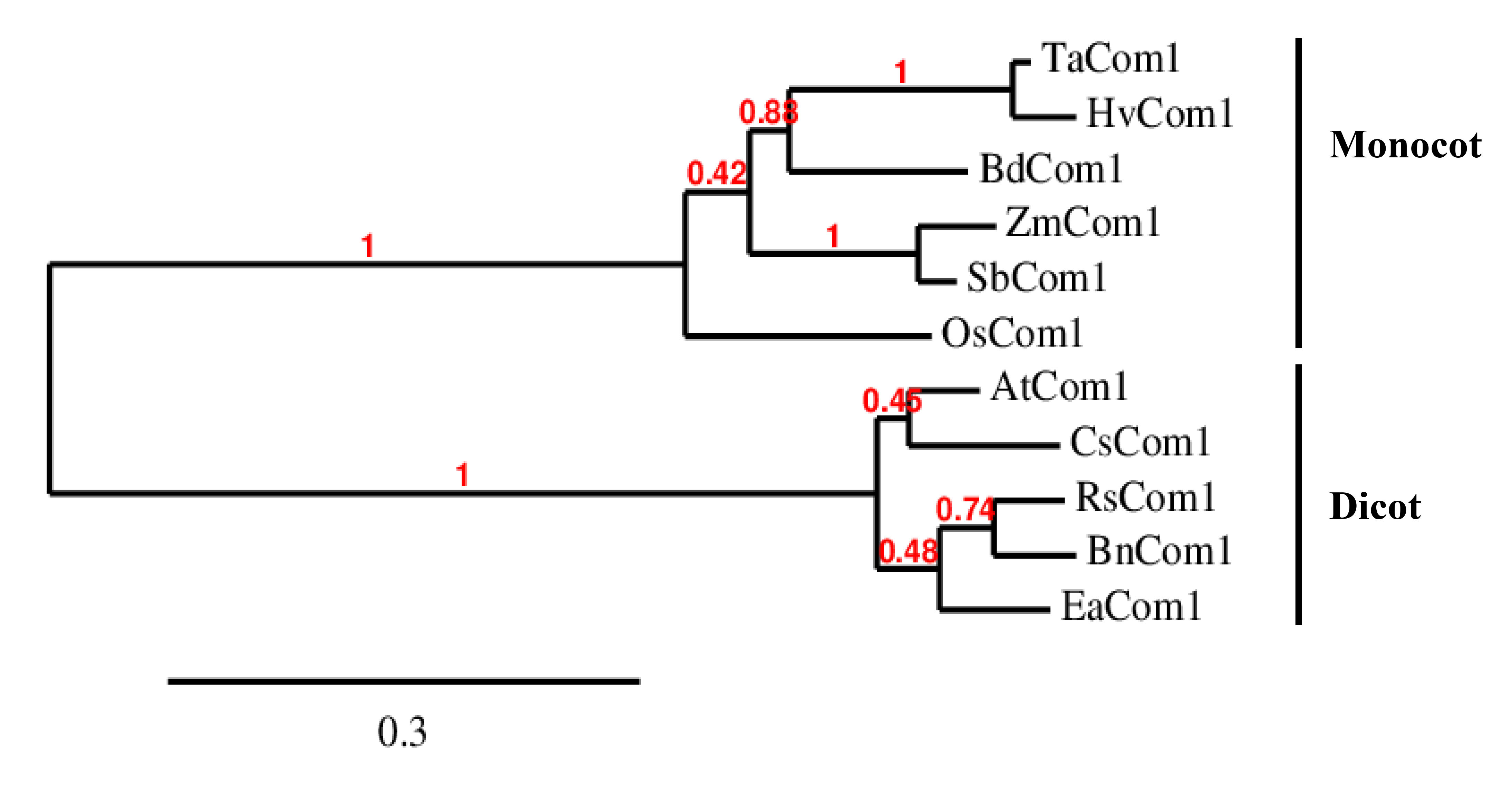

Supplement: FIGURE S2 — Neighbor-joining phylogeny reconstruction of Com1 homologs from different plant species. Numbers next to branches indicate posterior probability values. The scale indicates number of substitutions per site. Protein sequences were aligned using ClustalX (Jeanmougin et al., 1998) and phylogeny reconstruction was conducted using the online software (http://www.phylogeny.fr/, Dereeper et al., 2010). Species abbreviation: Zm, Zea mays; Os, Oryza sativa; Sb, Sorghum bicolor; At, Arabidopsis thaliana; Bn, Brassica napa; Bd, Brachypodium distachyon; Hv, Hordeum vulgare; Ta, Triticum aestivum; Cs, Camelina sativa; Rs, Raphanus sativus; Es, Eutrema salsugineum. [file Image_2.TIF]

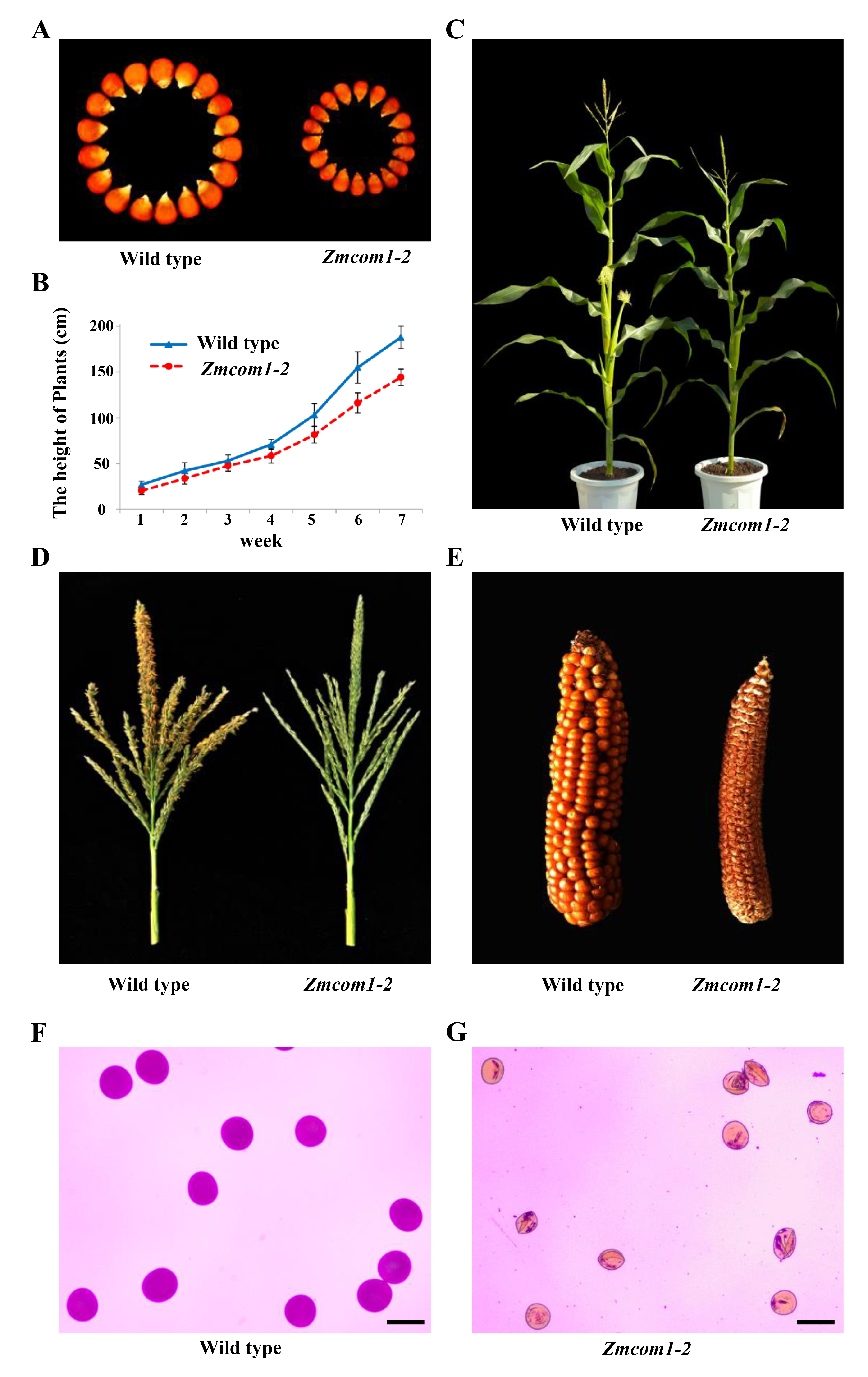

Supplement: FIGURE S3 — Morphological comparison between wild type and Zmcom1-2 mutant. (A) Morphological comparison of mature seeds between wild type and Zmcom1-2 mutant. (B) Growth-curve of plant height in wild type and Zmcom1-2 mutant plants. Values are means of 10 individual plants. (C) Comparison of Morphological comparison of mature plants between wild type and Zmcom1-2 mutant. (D) Comparison of a wild type tassel and a Zmcom1-2 tassel at the flowering stage. (E) Comparison of a wild type ear and a Zmcom1-2 ear. (F) Normal pollen grains of the wild type. Scale bar = 100 μm. (G) Complete sterile pollen grains of the Zmcom1-2 plant. Scale bar = 100μm. [file Image_3.TIF]

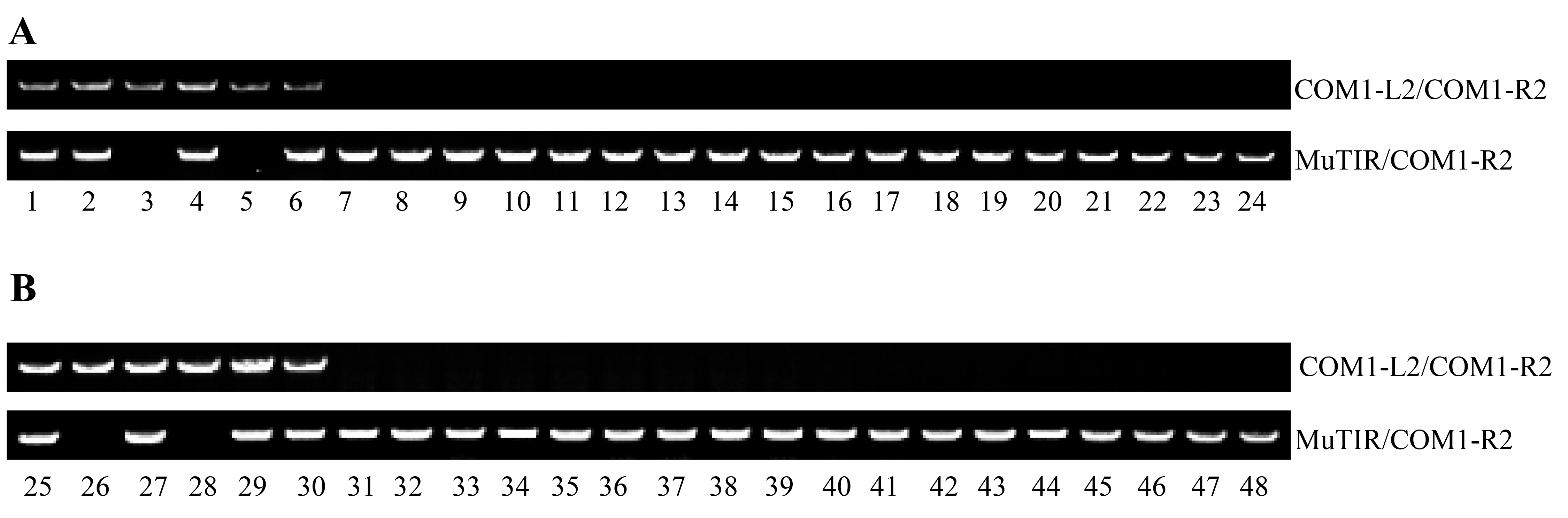

Supplement: FIGURE S4 — PCR-based genotyping of seeds from self-propagated heterozygous Zmcom1 plants. (A) F2 progeny of Zmcom1-1. 1–6: Seeds with normal size; 7–24: Seeds with small size. (B) F2 progeny of Zmcom1-2. 25–30: Seeds with normal size; 31–48: Seeds with small size. [file Image_4.TIF]

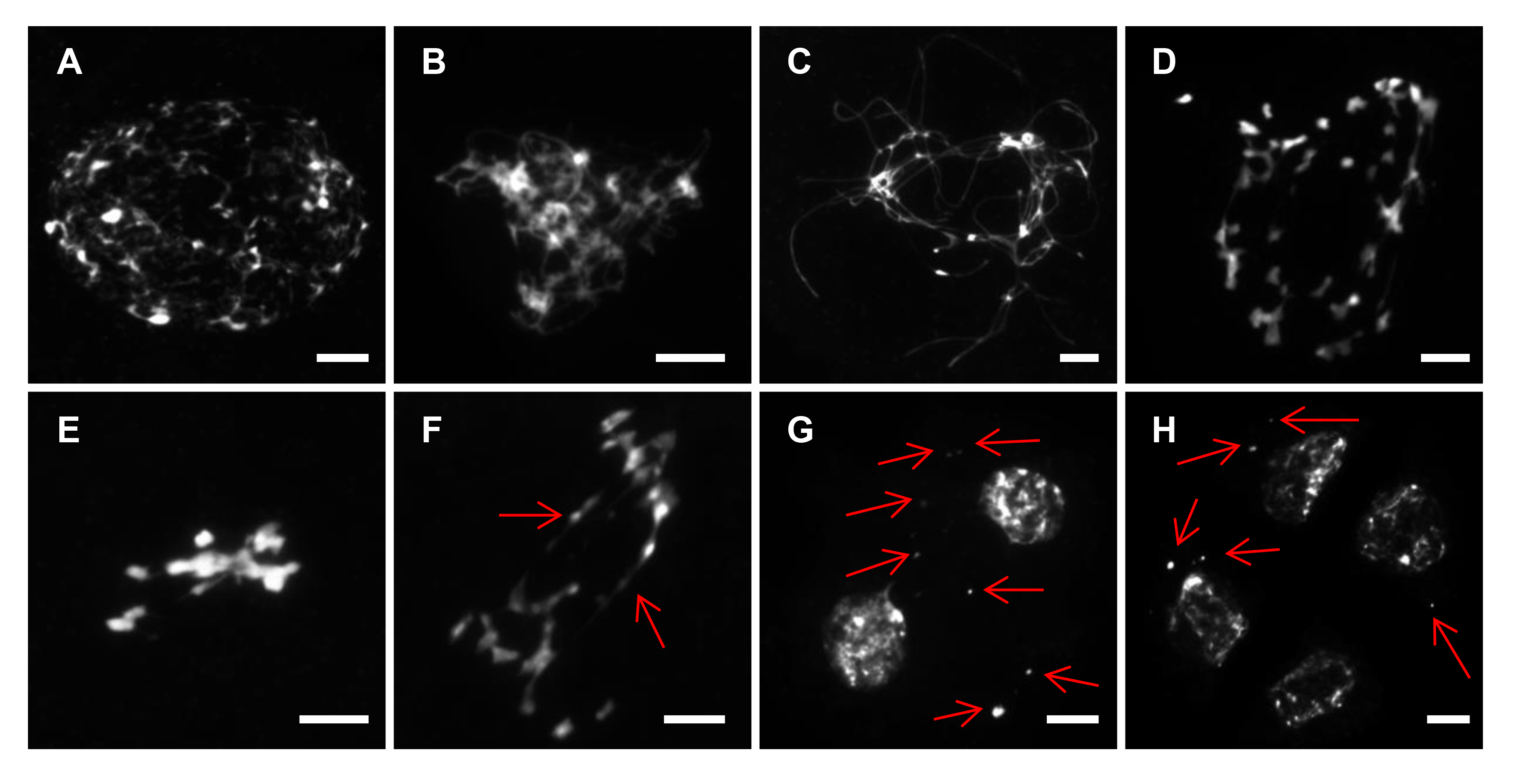

Supplement: FIGURE S5 — Male meiosis in Zmcom1-2. (A) Leptotene; (B) Zygotene; (C) Pachytene; (D) Diakinesis; (E) Metaphase I; (G) Anaphase I; (F) Dyad; (H) Tetrads. The red arrows pointed out the chromosomal fragments and abnormal bridges. Scale bars = 10 μm. [file Image_5.TIF]

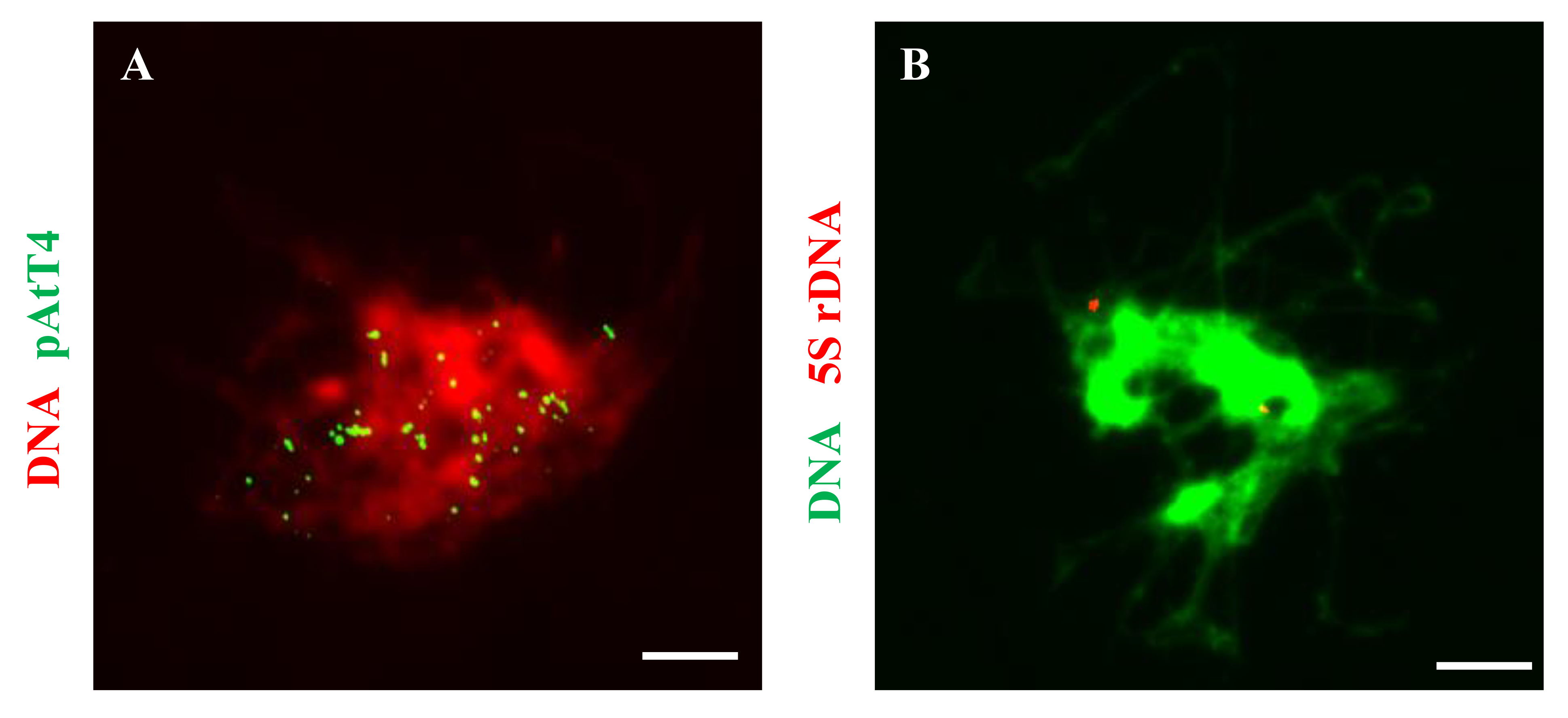

Supplement: FIGURE S6 — The defective bouquet formation and homologous pairing in Zmcom1-2. (A) Bouquet formation analysis using FISH with the telomere-specific pAtT4 probe in Zmcom1-2 (n = 41). Scale bars = 10 μm. (B) Homologous pairing analysis using FISH with 5S rDNA probe in in Zmcom1-2 (n = 27). Scale bars = 10 μm. [file Image_6.TIF]

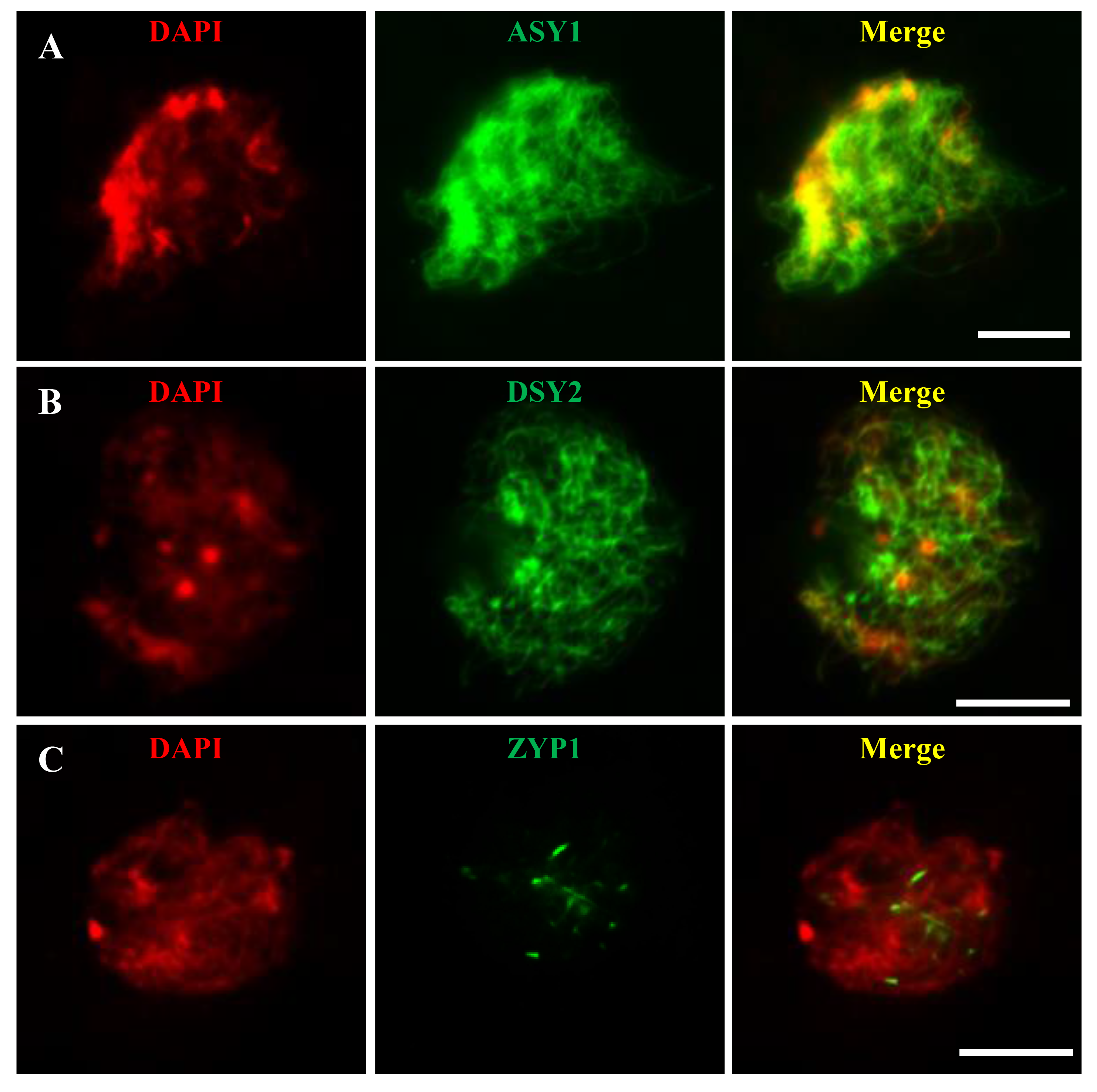

Supplement: FIGURE S7 — Immunolocalization of ASY1, DSY2, and ZYP1 antibodies in Zmcom1-2. ASY1 (A, n = 23), DSY2 (B, n = 33), and ZYP1 (C, n = 37) on prophase I chromosomes in Zmcom1-2. Scale bars = 10 μm. [file Image_7.TIF]

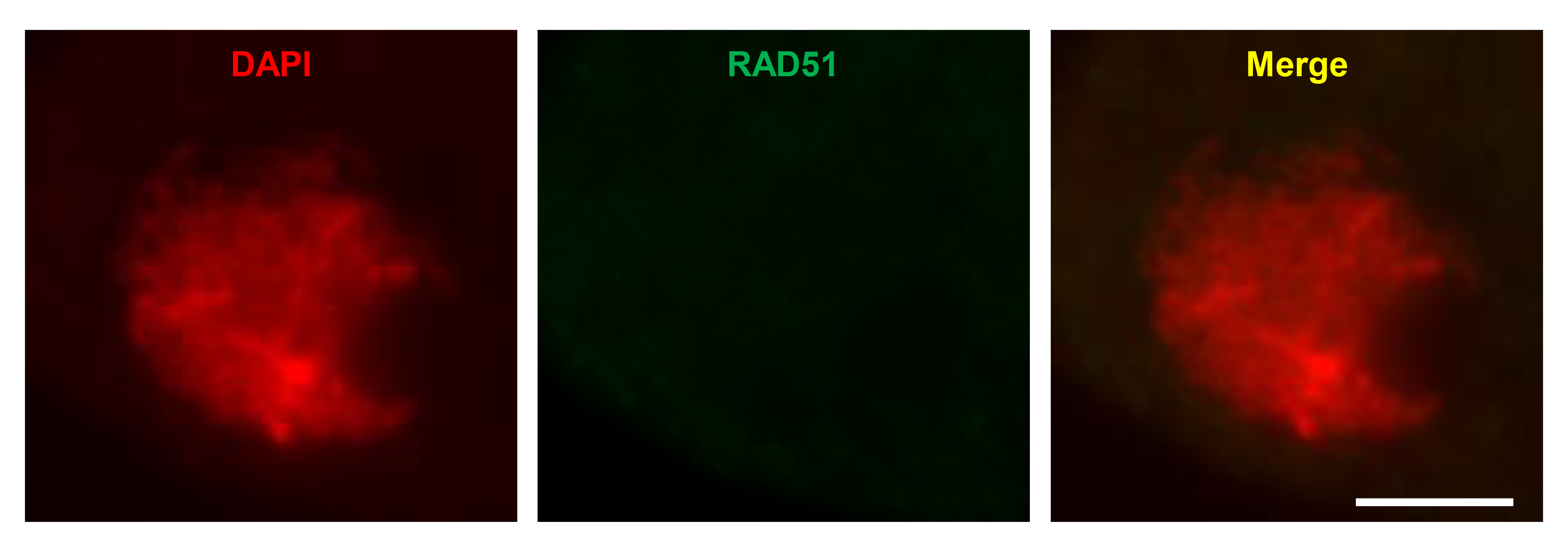

Supplement: FIGURE S8 — Immunolocalization of RAD51 antibodies in Zmcom1-2 meioctyes (n = 36) at zygotene. DAPI staining is used to indicate the chromosomes. Scale bars = 10 μm. [file Image_8.TIF]

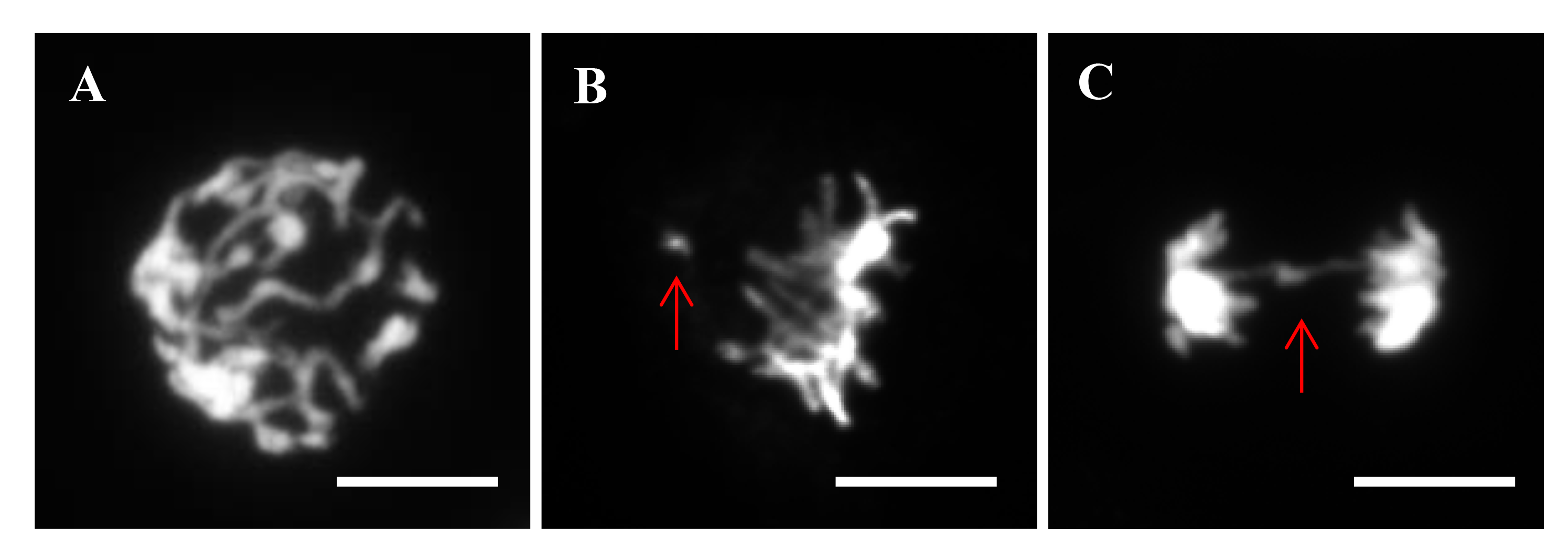

Supplement: FIGURE S9 — Genome instability in mitotic cells from Zmcom1-2 plants. (A) Prophase; (B) Metaphase; (C) Anaphase. Lagging chromosome fragments and anaphase bridges were highlighted by red arrows. Scale bars = 10 μm. [file Image_9.TIF]
