# Supplementary material for: ZmCom1 Is Required for Both Mitotic and Meiotic Recombination in Maize
Source: Front Plant Sci. 2018 Jul 16;9:1005. doi: 10.3389/fpls.2018.01005 (PMC6055016; doi:10.3389/fpls.2018.01005)
Supplement: TABLE S1 — Primers used in this study. [file Table_1.pdf]

Supplemental Table S1. Primers used in this study.

Prime name	Prime sequence（5'-3')	Purpose
COM1-L1	AGGTTGAGTGGAGGAAGACG	RT-PCR
COM1-R1	TCTTATCCTTCAGGTCAACCTG	
UBQ-L1	AAGATGCAGGCATCTAGGGCAAGG	
UBQ-R1	AGGCTCTTGGCTTGGCACATGTTC	
MuTIR	AGAGAAGCCAACGCCAWCGCCTCYATTTCGTC	Genotyping
COM1-L2	AGGTTGAGTGGAGGAAGACG	
COM1-R2	GACCCAAGTAGCATAAATCACG	
UBQ-L2	TAAGCTGCCGATGTGCCTGCG	RT-qPCR
UBQ-R2	CTGAAAGACAGAACATAATGAGCACA	
COM1-L3	CCATCCAGGAAGTGAAGGACCA	
COM1-R3	TTCATCGTGTCGGCGAGATG	
COM1-F	AGGCTACTGTTAGGATCATC	Cloning the CDS full-length of ZmCOM1 into pEASY Blunt Zero Vector
COM1-R	TCACAGTAGCTACTGCCTACCA	
PCUN-1F	GATGCTCACCCTGTTGTTTG	Examining the cellular localization
PCUN-1R	CAGATGAACTTCAGGGTCAGC	
PCUN-COM1-F	AAGCTTCACGTGGGCGCGCCGGATCATGGAGGGGAAGGCGGTA	
PCUN-COM1-R	CCCTTGCTCACCATGGTACCACTAGTCATTTCTGATTCGAAACCAA	
