# Supplementary material for: ZmCom1 Is Required for Both Mitotic and Meiotic Recombination in Maize
Source: Front Plant Sci. 2018 Jul 16;9:1005. doi: 10.3389/fpls.2018.01005 (PMC6055016; doi:10.3389/fpls.2018.01005)
Supplement: TABLE S2 — Segregation ratio of small seeds versus normal seeds. [file Table_2.pdf]

Table S2. Segregation ratio of small seeds versus normal seeds.

Parental genotype	Total No.of seeds from F2 progeny	No.of seed with small size	No.of seeds with normal size	X (1:3)[a]
Zmcom1-1/+ (Rep1)	132	30	102	0.14
Zmcom1-1/+ (Rep2)	165	40	125	0.02
Zmcom1-1/+ (Rep3)	186	43	143	0.13
Zmcom1-2/+ (Rep1)	154	35	119	0.16
Zmcom1-2/+ (Rep2)	143	32	111	0.20
Zmcom1-2/+ (Rep3)	195	45	150	0.14

[a]: χ^2 (0.05,1) = 3.84
